# Examining the Relationships Among Parental Overprotection, Military Life Adjustment, Social Anxiety, and Collective Efficacy

**DOI:** 10.3389/fpsyg.2021.613543

**Published:** 2021-02-11

**Authors:** Kyounghee Bark, Jung Hee Ha, Juliet Jue

**Affiliations:** ^1^Learning Science Department of Graduate School, Hanyang University, Seoul, South Korea; ^2^Graduate School of Counseling Psychology, Hanyang University, Seoul, South Korea; ^3^Department of Art Therapy, Hanyang Cyber University, Seoul, South Korea

**Keywords:** soldiers, parental overprotection, military life adjustment, social anxiety, collective efficacy

## Abstract

The purpose of this study was to verify the relationships among parental overprotection (PO), military life adjustment (MLA), social anxiety, and collective efficacy (CE). There have been studies examining the influence of each of these variables in isolation, but no study has examined these variables simultaneously. Two hundred and thirty-one male conscript soldiers participated in the study. Results indicated that all four variables were correlated with one another. Through hierarchical regression analysis, we determined that social anxiety fully mediated the relationship between PO and MLA. Furthermore, we found that CE moderated the relationship between PO and social anxiety. Finally, we confirmed the moderated mediation effect of CE in our proposed model. We discuss the implications and limitations of this model.

## Introduction

Military life adjustment (MLA) is an adaptation process in relation to the military environment. It can be characterized as having a sense of duty to one’s mission as well as feeling contentment with military life – that is, being satisfied with one’s position and duties (Stauffer et al., 1949).

In Korea, all men in their early twenties are obligated to serve as soldiers under the Military Service Act (Ministry of National Defense, 2018). Due to involuntary enlistment, conscript soldiers often experience low motivation or even adjustment problems in military service (Koo, 2005, 2013). Maladjustment within the military may lead to accidents or early discharge of soldiers, so it is important to explore the variables that affect soldiers’ adjustment.

The environmental factors that influence MLA can be divided into three key categories: unit, personal, and family factors. Regarding unit factors, extensive research has been conducted on military life stress as a major influence on MLA (Hyun and Lee, 2008; Lee and Ra, 2013; Kim et al., 2014).

Military systems and training programs have important effects on soldiers’ adaptation, but soldiers’ individual characteristics have also been found to be influential (Lee, 2018). Accordingly, several studies have focused on individual soldiers’ psychological characteristics and identified the following factors that influence MLA: stress coping methods (Park and Jeon, 2013), resilience (Sim and Kim, 2013), mental health (Joe, 2003; Kim and Hah, 2013; Park and Lee, 2014), self-complexity (Sim and Kim, 2013), and social support (Lee and Ra, 2013; Lee and Kim, 2015). Furthermore, more recent research has focused on the third factor mentioned above; namely, the family factor (Kwon and Kim, 2013; Lee and Cho, 2015; Cho, 2020). In a study of soldiers’ adjustment to military life, 32% of maladjusted soldiers reported having problems with family relationships (Kim et al., 2014). This result suggests that factors such as the influence of family should be considered in soldiers’ MLA.

Being around the age of 20, conscript soldiers have a physical age corresponding to adulthood, but they are economically dependent on their parents and likewise have not achieved sufficient psychological independence; in this regard, they are in the midst of a unique life stage that exists separately between adolescence and adulthood (Arnett, 2000). Thus, recent studies on military adaptation have focused on the impact of soldiers’ family environments and their psychological maturity.

Parenting styles – as reported by children – contain much information about how children interact with their parents. Parenting styles affect children’s emotional and cognitive development (McLeod et al., 2007; Ingen et al., 2015) and psychosocial adaptation (Barber and Harmon, 2002; Wolfradt et al., 2003; Jo and Chong, 2017). Parenting style impacts childhood and adolescence considerably, but its influence also persists into adulthood (Cho and Choi, 2015).

Parental overprotection (PO) refers to a parenting style characterized by exerting excessive control, limiting independent behavior, and treating children as younger than their actual age (Levy, 1941). PO has been found to affect children’s adaptation negatively (Kim and Yang, 2018). It inhibits children’s psychosocial development, and it may lead to psychopathology and neurosis (Adler, 1931), as well as the atrophy of autonomy by limiting children’s experiences (Hudson and Rapee, 2001; Barber and Harmon, 2002; Ingen et al., 2015). These children often experience social anxiety (LeMoyne and Buchanan, 2011; Borelli et al., 2015) or present problems such as low school-life satisfaction, low academic achievement, substance abuse, and feelings of incompetence (Schiffrin et al., 2014; Nelson et al., 2015). Jeong and Kim (2016) report that soldiers who have experienced PO are more susceptible to adjustment issues in the military than are those who have not experienced this overprotection.

Parental overprotection may cause social anxiety (Kim et al., 2005; Suh et al., 2010). Rapee and Melville (1997) noticed in their study that patients with social anxiety disorder tended to report that their parents were overprotective, and later studies also found high correlations between social anxiety and PO (Arim and Shapka, 2008; Festa and Ginsburg, 2011). Additionally, people with social anxiety have low expectations about their ability to cope with social situations (Kachin et al., 2001; Ingen et al., 2015), and low overall levels of self-efficacy are related to high levels of social anxiety (Lancu et al., 2015).

Collective efficacy (CE) also is one of the major factors influencing individual adaptation. CE may be defined as a group member’s perception that the group is able to complete a specific task (Bandura, 2000) or as the group member’s confidence in the group’s effective achievement of goals and the successful performance of specific tasks (Zellars et al., 2001; Blecharz et al., 2014). CE reduces burnout and job stress and facilitates work effectiveness, motivation, goal achievement, job satisfaction, and adjustment (Bandura, 2000; Sejits et al., 2000; Zellars et al., 2001; Gully et al., 2002; Hochwarter et al., 2003; Roos et al., 2013; Blecharz et al., 2014). Studies with military soldiers have demonstrated that higher CE is associated with better MLA (Borgogni et al., 2010; Kwon and Kim, 2013) and higher job satisfaction (Borgogni et al., 2010). Previous studies found a negative relationship between soldiers’ self-reported degree of PO and self-efficacy (Kim et al., 2005), as well as a negative relationship between self-efficacy and social anxiety (Thomasson and Psouni, 2010).

**Figure 1:**
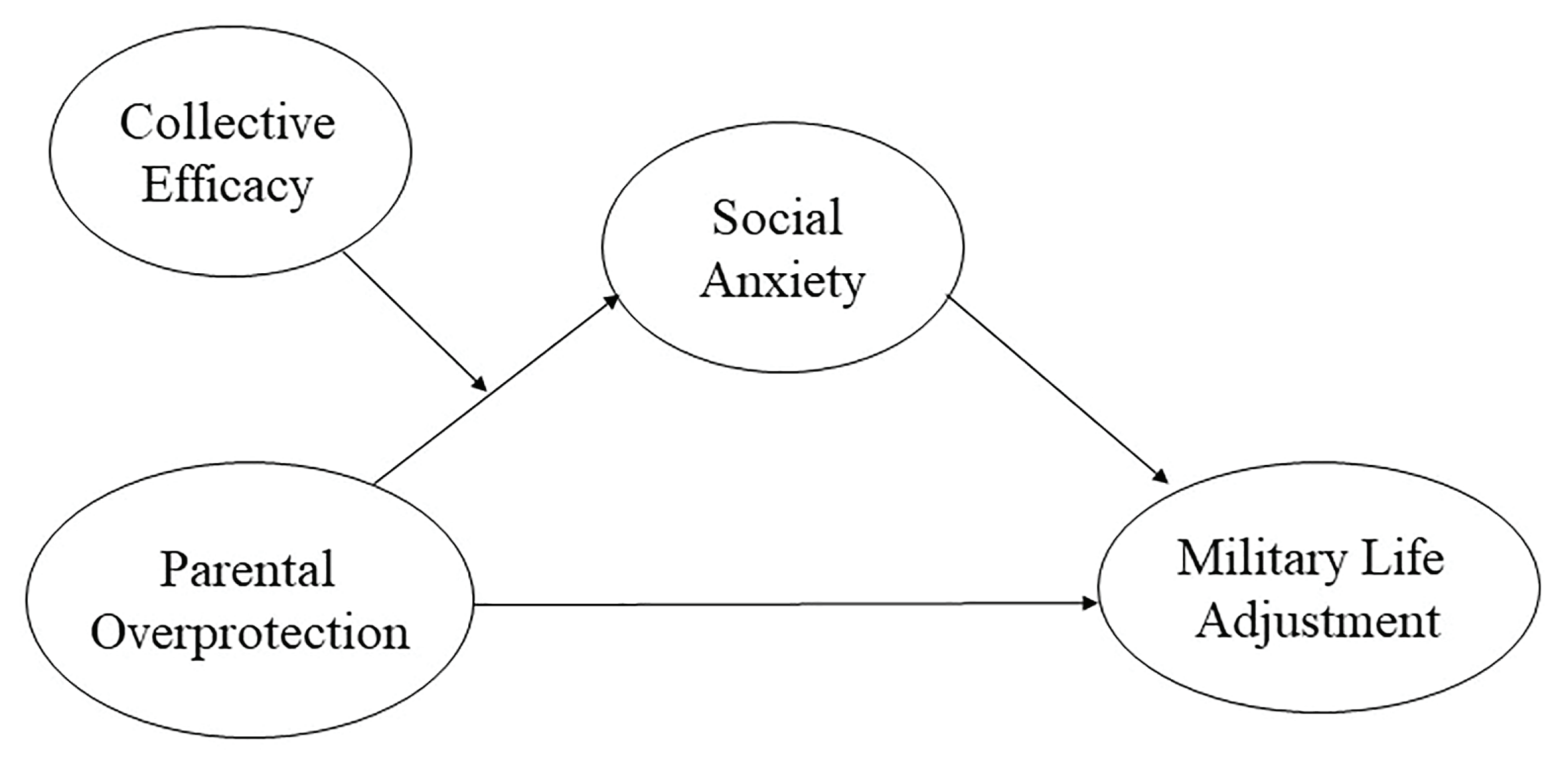
Hypothesized model of the relationships among parental overprotection, social anxiety, collective efficacy, and military life adjustment.

Based on these previous studies, we sought to examine the relationship between PO and MLA while accounting for the roles of social anxiety and CE (see Figure 1). Past studies have examined the influence of each of these variables in isolation, but no study has examined all variables simultaneously. Thus, we aimed to test the validity of this more inclusive model in this study.

## Materials and Methods

### Participants

We collected data by distributing questionnaires to soldiers in the Korean Army. A total of 265 individuals participated in the survey. We excluded 34 incomplete responses from analysis, thus utilizing data from 231 respondents. All participants were male, and their average age was 21.09years (*SD* = 0.83). The age distribution was as follows: 38.1% were age 22 or older, 33.3% were age 21, 27.7% were age 20, and 0.9% were age 19 or younger. As for class, 24.7% had the rank of sergeant, 37.2% had the rank of corporal, 37.2% had the rank of private first class, and 0.9% had the rank of private. The distribution of educational background was as follows: 19.5% were high school graduates, 74.9% were in college, and 5.7% were college graduates.

### Procedures

Soldiers’ participation was voluntary, and all participants provided informed consent before starting the survey. All data were kept anonymous. Participants received a small gift for completing the questionnaire.

### Measures

#### Parental Overprotection Scale

To measure PO, we used the PO scale developed by Huh (2004). The original scale includes eight subscales: (1) supervision, (2) rational explanation, (3) inconsistency, (4) excessive expectation, (5) excessive interference, (6) abuse, (7) neglect, and (8) affection. We used only the excessive expectation (six items) and excessive interference (seven items) subscales in this study. Items were rated on a four-point Likert scale ranging from 1 (*not at all likely*) to 4 (*extremely likely*). The scale demonstrated excellent internal consistency in this study (Chronbach’s *α* = 0.93).

#### Social Interaction Anxiety Scale (SIAS) – Korean Version

The SIAS was developed by Mattick and Clarke (1998) and was translated to Korean by Kim (2001, Unpublished). This scale comprises 20 items measuring cognitive, emotional, and behavioral responses to social interactions, such as meeting and socializing with new people. Items were rated on a five-point Likert scale ranging from 0 (*not at all likely*) to 4 (*extremely likely*). Higher total scores represent higher levels of anxiety and fear in social interactions. The scale demonstrated excellent internal consistency both in the original authors’ study (*α* = 0.93) and in this study (*α* = 0.96).

#### Military Life Adjustment Scale

To measure adjustment to military life, we employed the MLA scale developed by Stauffer et al. (1949). It was translated and modified by Shin (1981, Unpublished) to address the Korean military, specifically. In this study, we used 20 items from the scale representing four domains: (1) stability of mind and body (three items), (2) willingness to perform the assigned mission (four items), (3) job satisfaction (five items), and (4) positive attitude toward the military organization (eight items). Items were rated on a five-point Likert scale ranging from 0 (*not at all likely*) to 4 (*extremely likely*). Higher total scores indicate better MLA. The scale demonstrated excellent internal consistency in this study (*α* = 0.94).

#### Collective Efficacy Scale

We used the CE scale developed by Kwon and Kim (2016) to evaluate soldiers’ CE. This scale consists of 18 items spanning four factors: (1) commanders’ ability (five items), (2) troop members’ ability (five items), (3) unit’s physical environment (five items), and (4) unit’s atmosphere (three items). Items were rated on a five-point Likert scale ranging from 0 (*not at all likely*) to 4 (*extremely likely*). Higher total scores reflect greater CE. The scale demonstrated excellent internal consistency in the original authors’ study and in this study (*α* = 0.95 in both cases).

### Data Analysis

We used SPSS Statistics 22.0 and the SPSS PROCESS macro (Hayes, 2012) to analyze the data. After performing descriptive statistics and correlational analysis, we verified the mediating effect of the main variable. In addition, we conducted bootstrapping to verify the significance of the mediating effect. To verify the moderated mediation effect, we examined the presence of the moderating effect of the main variable (1) in the relationship between the independent variable and the dependent variable, as well as (2) in the relationship between the independent variable and the mediating variable. Additionally, we confirmed the simple slope of the moderating effect.

## Results

### Descriptive Statistics and Correlational Analysis of Variables

As presented in Table 1, we found several significant correlations among the main variables. PO was correlated positively with social anxiety (*r* = 0.59, *p* < 0.001) yet was correlated negatively with MLA (*r* = −0.29, *p* < 0.001) and with CE (*r* = −0.28, *p* < 0.001). Social anxiety was correlated negatively with CE (*r* = −0.46, *p* < 0.001) and with MLA (*r* = −0.49, *p* < 0.001). CE, however, was correlated positively with MLA (*r* = 0.72, *p* < 0.001).

**Table 1:** Correlation coefficients and descriptive statistics for measurement variables.

	Parental overprotection	Social anxiety	Collective efficacy	Military life adjustment
Parental overprotection	–			
Social anxiety	0.59^***^	–		
Collective efficacy	−0.28^***^	−0.46^***^	–	
Military life adjustment	−0.29^***^	−0.49^***^	0.72^***^	–
Mean	2.00	1.44	2.66	2.50
SD	0.63	0.89	0.80	0.76
Skewness	0.03	0.27	0.14	−0.14
Kurtosis	−0.74	−0.86	−0.90	−0.12

*** *p* < *0.001.*

Table 1 also presents the skewness and kurtosis of the main variables. Among the measurement variables in this study, the largest absolute value of skewness was 0.27, and the largest absolute value of kurtosis was −0.90, which satisfied the normality assumption.

### Mediating Effect Verification

We performed the hierarchical regression analysis proposed by Baron and Kenny (1986) to verify the assumption that PO affects MLA through social anxiety. Table 2 presents the results of this analysis.

**Table 2:** Mediating effect of social anxiety.

Step	Independent variable	Dependent variable	*B*	*SE*	*β*	*R^2^*	*F*
Step 1	PO	MLA	−0.54	0.12	−0.29	0.08	21.01^***^
Step 2	PO	Social anxiety	1.30	0.12	0.60	0.36	127.06^***^
Step 3	PO	MLA	0.01	0.13	0.01	0.24	36.72^***^
	Social anxiety		−0.42	0.06	−0.50		

*** *p* < *0.001.*

PO, Parental overprotection; MLA, Military life adjustment.

In the first stage, we found that PO significantly predicted MLA (*β* = −0.29, *p* < 0.001). In the second stage, PO significantly predicted social anxiety (*β* = 0.60, *p* < 0.001). In the third stage, PO and social anxiety were used simultaneously as independent variables, which significantly predicted MLA (*β* = −0.50, *p* < 0.001). The absolute value of the standardized coefficient (*β*) of PO was significant in the first stage (*β* = −0.29, *p* < 0.001), but not in the third stage (*β* = 0.01, *p* = 0.92). Therefore, social anxiety had a complete mediating effect on the relationship between PO and MLA.

We conducted bootstrapping using PROCESS to verify the statistical significance of the mediating effects. The number of samples reextracted was 5,000, and the mediating effect coefficient was −0.55. As presented in Table 3, the confidence interval lower limit (LLCI) and upper limit (ULCI) values of the mediating effect coefficient were −0.79 and −0.35, respectively. Because this interval does not include zero (Preacher and Hayes, 2004), we concluded that the mediating effect of anxiety was statistically significant.

**Table 3:** Bootstrapping results for the mediating effect of social anxiety.

Variable	Mediating effect coefficient	Boot SE	95% Confidence interval	
			Boot LLCI	Boot ULCI
Social anxiety	−0.55	0.11	−0.79	−0.35

*SE, standard error; LLCI, Lower limit confidence interval; ULCI, Upper limit confidence interval*.

### Moderating Effect Verification

We examined the moderating effect of CE in the relationship between PO and social anxiety using a hierarchical regression analysis procedure (Aiken and West, 1991). To minimize multicollinearity, the independent variables and the control variable were analyzed after mean-centering (Cohen et al., 2003). We confirmed the significance of the interaction term and the additional explanatory amount of the hierarchical regression analysis. Additionally, we used PROCESS to confirm the statistical significance of the simple regression line that appeared for each moderating effect. Table 4 presents the results of the analysis.

**Table 4:** Moderating effect of collective efficacy on social anxiety.

	Dependent variable: Social anxiety						
	Unstandardized coefficients		*β*	*t*	*R^2^*	∆*R^2^*	∆*F*
	*B*	*SE*					
Step 1							
(Constant)	1.45	0.04		33.06^***^	0.45	0.45	93.57^***^
PO (A)	0.72	0.07	0.51	9.88^***^			
CE (B)	−0.35	0.06	−0.32	−6.25^***^			
Step 2							
(Constant)	1.41	0.04		31.75^***^	0.48	0.03	11.70^***^
PO (A)	0.72	0.07	0.51	10.13^***^			
CE (B)	−0.37	0.06	−0.33	−6.57^***^			
A × B	−0.27	0.08	−0.16	−3.42^**^			

** *p* < *0.01;*

*** *p* < *0.001.*

PO, Parental overprotection; CE, Collective efficacy.

**Figure 2:**
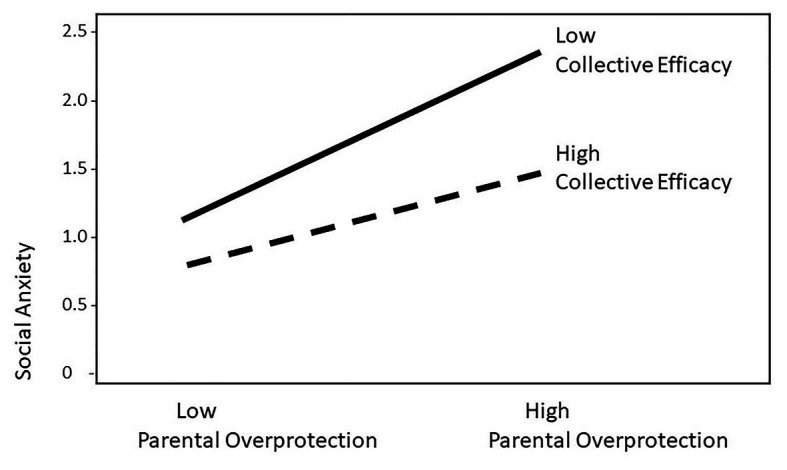
Interaction effect of parental overprotection and collective efficacy. All estimates are standardized coefficients.

We found that the interaction of PO and CE had a significant effect on social anxiety (*β* = −0.16, *p* < 0.01) and that its incremental volume of *R^2^* was significant (∆*R^2^* = 0.03, ∆*F* = 11.70). Thus, CE significantly moderates the relationship between PO and social anxiety. Additionally, we reconfirmed the moderating effect of CE by checking the graphs: We divided responses on the independent variable and the control variable into upper and lower groups, respectively, and confirmed the moderating effect using a graph that induces a regression equation (see Figure 2).

Next, we verified the statistical significance of the simple regression line with the modulatory variable at a specific condition value (mean, mean ± 1 *SD*) in order to confirm the moderating effect pattern (Aiken and West, 1991). When the level of CE was low (−1 *SD*), there was a statistically significant effect of PO on social anxiety (*B* = 0.94, *t* = 9.76, *p* < 0.001). When CE was at the average level, the effect of PO still was significant (*B* = 0.72, *t* = 10.13, *p* < 0.001). Likewise, when the level of CE was high (+1 *SD*), the effect was significant (*B* = 0.50, *t* = 5.21, *p* < 0.01). Therefore, we confirmed that this effect was statistically significant in all cases.

### Verification of Prerequisites for Moderated Mediation Effect

To determine whether the prerequisites of the moderated mediation effect were satisfied, we first tested the moderating effect of CE in the relationship between PO and MLA. To limit multicollinearity, the independent variable and the control variable were analyzed after mean-centering (Cohen et al., 2003). The results showed that the interaction term of PO and CE was not statistically significant (*β* = 0.02, *p* > 0.05); likewise, its incremental volume of *R^2^* was not significant (∆*R^2^* = 0.00, ∆*F* = 0.10). This confirmed that CE did not control PO and MLA, which satisfied the preceding basic assumption of the moderated mediation effect.

### Moderated Mediation Effect Verification

**Figure 3:**
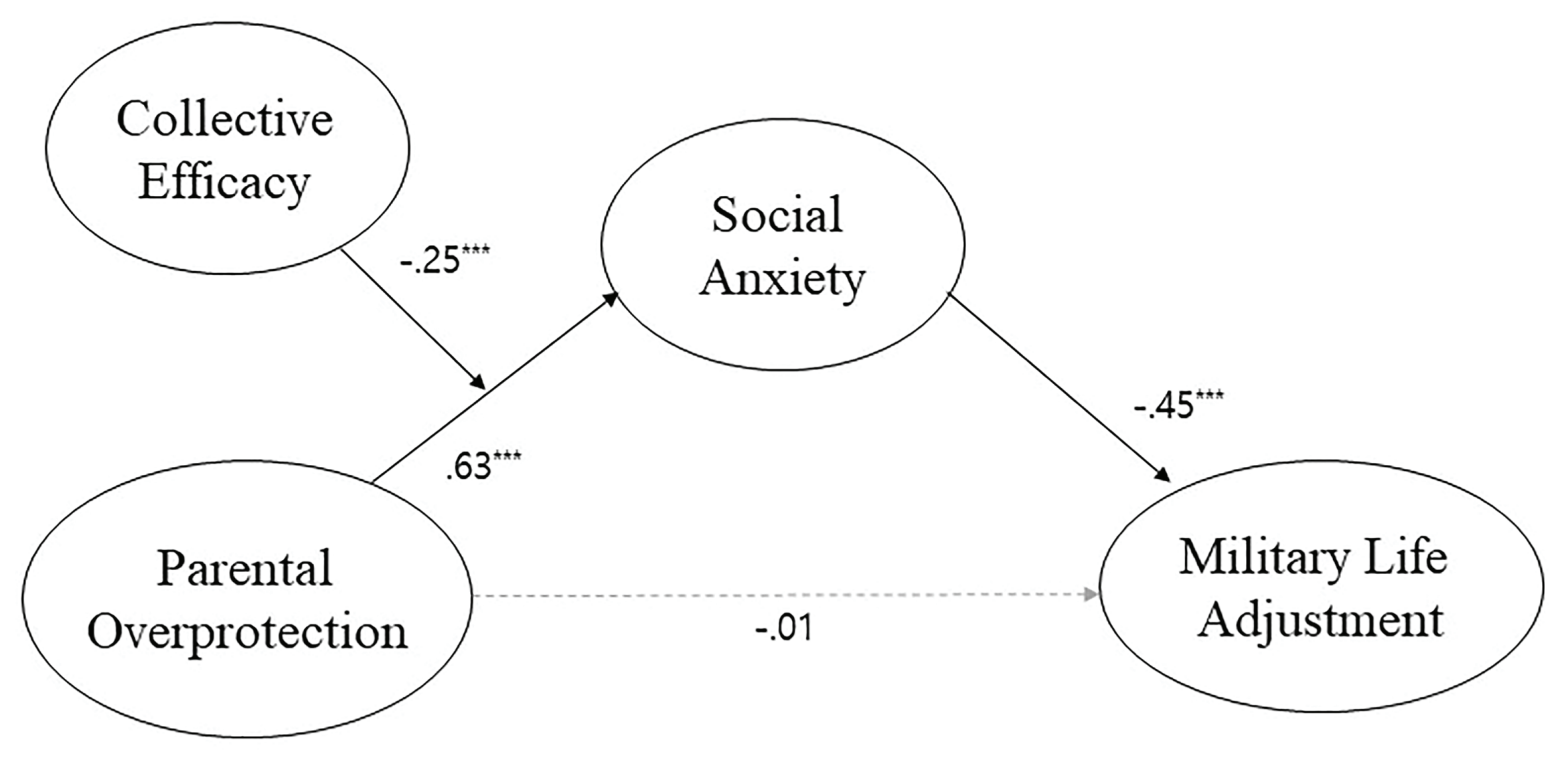
Final model of the relationships among parental overprotection, social anxiety, collective efficacy, and military life adjustment. ^***^p < 0.001.

We analyzed the moderated mediation effect of CE in the relationship between PO and social anxiety using PROCESS (Hayes, 2012). Figure 3 presents the moderated mediation effect model, and Table 5 displays the results of the analysis. We found that the interaction between PO and CE affected social anxiety (*B* = −0.25, *p* < 0.001), which subsequently affected MLA (*B* = −0.45, *p* < 0.001).

**Table 5:** Moderated mediation effect of collective efficacy.

Dependent variable: Social anxiety					
	*B*	*SE*	*t*	LLCI	ULCI
(Constant)	1.49	0.04	38.38^***^	1.41	1.56
PO (A)	0.63	0.06	10.23^***^	0.51	0.76
Collective efficacy (B)	−0.30	0.05	−6.21^***^	−0.40	−0.21
A X B	−0.25	0.07	−3.61^***^	−0.39	−0.11
Dependent variable: Military life adjustment					
	*B*	*SE*	*t*	LLCI	ULCI
(Constant)	3.20	0.12	27.34^***^	2.97	3.43
PO	−0.01	0.09	−0.14	−0.19	0.16
Social anxiety	−0.45	0.07	−6.39^***^	−0.59	−0.31

*** *p* < *0.001.*

PO, Parental overprotection; LLCI, Lower limit confidence interval; ULCI, Upper limit confidence interval.

To verify the magnitude and direction of the conditional indirect effect – which is the change depending on the level of the moderating variable – we used the bootstrapping method. Table 6 presents these results. The moderated mediation effect was significant in all three levels (mean, mean − 1 *SD*, mean + 1 *SD*).

**Table 6:** Conditional indirect effect of collective efficacy.

Variable	Condition	Effect	Boot SE	95% Confidence interval	
				Boot LLCI	Boot ULCI
Social anxiety	−1 *SD*	−0.38	0.07	−0.53	−0.25
	M	−0.29	0.06	−0.42	−0.18
	+1 *SD*	−0.20	0.06	−0.34	−0.09

*SE, standard error; LLCI, Lower limit confidence interval; ULCI, Upper limit confidence interval*.

## Discussion

The purpose of this study was to investigate the relationship between PO, social anxiety, MLA, and CE. Our study produced four major findings.

First, we confirmed several variables’ relationships that were found in past research. We found that PO was correlated negatively with MLA, which aligns with previous findings (Barber and Harmon, 2002; Kim and Yang, 2018; Cho, 2020). PO impairs children’s interpersonal relationships and hinders later adaptation to early adulthood (Hudson and Rapee, 2001; Barber and Harmon, 2002; Jeong and Kim, 2016). Another study found that negative parenting hinders adult children’s MLA (Cho, 2020). Therefore, one might suggest that a soldier’s degree of autonomy, which has been degraded due to PO, becomes relatively worse within the military environment, causing anxiety, depression, and difficulty adjusting to military life.

The fact that PO was correlated positively with social anxiety supports findings from previous studies examining the relationship between parental attitudes and social anxiety (Barber and Harmon, 2002; Wolfradt et al., 2003; Arim and Shapka, 2008; Suh et al., 2010). Kim and Yang (2018) contend that if overprotective parenting continues, children will depend on their parents even after entering adulthood and will experience social anxiety due to feelings of inferiority.

We found that social anxiety was negatively correlated with MLA. This also aligns with previous findings (Mather et al., 2010). Researchers insist that social anxiety can have serious consequences, including avoidance of social situations, school rejection, substance abuse, and even suicide (Stein and Gorman, 2001; Ameringen et al., 2003; Costello et al., 2003). People with high levels of anxiety use self-protective communication strategies, such as communicating less frequently and expressing less intimate content than others (Kachin et al., 2001). If soldiers with high levels of social anxiety use similar communication styles, they might face difficulties in their relationships with fellow soldiers, which could result in military life maladjustment.

In this study, CE was correlated negatively with PO and with social anxiety. In a similar vein, Givertz and Segrin (2012) observe that higher levels of PO tend to be associated with lower levels of self-efficacy for children. Moreover, self-efficacy has been reported to have a negative correlation with social anxiety (Neal and Edelman, 2003; Kim et al., 2005; Lancu et al., 2015). On the contrary, CE was correlated positively with MLA (Borgogni et al., 2010; Kwon and Kim, 2013), which supports previous findings regarding the relationships among CE, organizational commitment, and MLA (Kwon, 2016, Unpublished). This result implies that the belief in fellow soldiers’ ability and performance influences each soldier positively and subsequently affects MLA.

Second, we found that social anxiety has a complete mediation effect in the relationship between PO and MLA. This result supports previous findings that parenting styles characterized by excessive control and meddling tend to increase children’s anxiety (Suh et al., 2013; Borelli et al., 2015), parent-child conflicts, and overall psychological distress, all of which affect adjustment (Hudson and Rapee, 2001; LeMoyne and Buchanan, 2011; Schiffrin et al., 2014; Nelson et al., 2015). Additionally, increased childhood anxiety may lead to social withdrawal and anxiety symptoms in adulthood (Spokas et al., 2009; Knappe et al., 2012).

Third, we confirmed that CE moderates the relationship between PO and social anxiety. When CE was high, PO had a small effect on social anxiety. This result can be explained by the fact that self-efficacy is closely related to social anxiety (Lancu et al., 2015), and there was a negative correlation between them (Thomasson and Psouni, 2010). Based on these findings, we contend that in order to enhance CE, the military should do more to enhance the commanders’ leadership (Sosik and Godshalk, 2000; Kark et al., 2003), to facilitate soldiers’ interactions with commanders, and to establish trust among soldiers (Koo, 2005), which could diminish soldiers’ social anxiety.

Fourth, we confirmed the moderated mediation effect of CE in our proposed model. The mediating effect varies depending on the moderating variable; in our study, the relationship between PO and social anxiety varied depending on the level of CE. Although PO affects social anxiety, soldiers with high CE could lower their social anxiety and subsequently enhance their MLA. CE can be formed through experiencing group success, positive leadership climate (Chen and Bliese, 2002), and constructive interactions with fellow soldiers (Watson et al., 2001). Thus, these experiences are imperative, as they may lower soldiers’ social anxiety of soldiers, which, in turn, may have a positive effect on their MLA.

This study validated a comprehensive model that examines the complex factors affecting soldiers’ adjustment to military life. We examined parenting factors, which can affect individual psychological growth, in relation to MLA. There have been studies examining MLA, but these studies often have overlooked the important influence of parenting factors because of their treatment of soldiers as independent adults. Despite being in their early twenties, most soldiers exhibit psychological regression due to the special conditions of the unfamiliar military organization as well as due to the process of conscription. Therefore, we argue that it is necessary to examine the impact of parenting in relation to a military adaptation.

Based on our findings, we propose that interventions to improve the MLA of soldiers who were overprotected by their parents should focus on reducing social anxiety and enhancing CE. Even if young people who grew up in a setting of overprotection are vulnerable, they can adapt to military life more easily when their social anxiety is reduced. Thus, implementing educational programs focused on reducing anxiety would be beneficial to soldiers. Moreover, as the study has revealed, CE plays a moderating role – this means that it is necessary to consider various methods to lower social anxiety by increasing group efficacy. Specifically, it would be possible to increase the sense of CE through commanders’ qualified leadership and by facilitating fellowship and attachment among colleagues. This would lower soldiers’ social anxiety and improve their MLA.

This study has two main limitations. First, all participants were male, Korean conscript soldiers. Due to these characteristics, the results may not generalize to all soldiers. Future studies may benefit from surveying volunteer soldiers and female soldiers. Second, the SIAS captures only one type of anxiety: social anxiety. Future studies may benefit from using another scale to evaluate performance anxiety and comparing the results with those of this study.

## Data Availability Statement

The datasets presented in this article are not readily available due to the nature of this research, participants of this study did not agree for their data to be shared publicly, so supporting data is not available. Requests to access the datasets should be directed to JH, hajung366@hanyang.ac.kr.

## Ethics Statement

Ethical review and approval was not required for the study on human participants in accordance with the local legislation and institutional requirements. The patients/participants provided their written informed consent to participate in this study.

## Author Contributions

KB and JH conceived the presented idea, developed the theory, and analyzed the data. KB conducted the survey. JH verified the analytical methods. KB and JJ wrote the manuscript in consultation with JH. All authors contributed to the article and approved the submitted version.

### Conflict of Interest

The authors declare that the research was conducted in the absence of any commercial or financial relationships that could be construed as a potential conflict of interest.
